# Fundamental Issues Related to the Origin of Melatonin and Melatonin Isomers during Evolution: Relation to Their Biological Functions

**DOI:** 10.3390/ijms150915858

**Published:** 2014-09-09

**Authors:** Dun-Xian Tan, Xiaodong Zheng, Jin Kong, Lucien C. Manchester, Ruediger Hardeland, Seok Joong Kim, Xiaoying Xu, Russel J. Reiter

**Affiliations:** 1 Department of Cellular and Structural Biology, the University of Texas, Health Science Center, San Antonio, TX 78229, USA; E-Mails: lmanchester@stmarytx.edu (L.C.M.); skim@dongduk.ac.kr (S.J.K.); xxy2006stone@163.com (X.X.); 2 Institute for Horticultural Plants, China Agricultural University, Beijing 100083, China; E-Mails: zheng.xiao.d@163.com (X.Z.); 05021@cau.edu.cn (J.K.); 3 Johann Friedrich Blumenbach Institute of Zoology and Anthropology, University of Göttingen, Göttingen 37073, Germany; E-Mail: rhardel@gwdg.de

**Keywords:** melatonin, isomer, archaea, cyanobacteria, mitochondria, chloroplasts, plants, antioxidant, evolution

## Abstract

Melatonin and melatonin isomers exist and/or coexist in living organisms including yeasts, bacteria and plants. The levels of melatonin isomers are significantly higher than that of melatonin in some plants and in several fermented products such as in wine and bread. Currently, there are no reports documenting the presence of melatonin isomers in vertebrates. From an evolutionary point of view, it is unlikely that melatonin isomers do not exist in vertebrates. On the other hand, large quantities of the microbial flora exist in the gut of the vertebrates. These microorganisms frequently exchange materials with the host. Melatonin isomers, which are produced by these organisms inevitably enter the host’s system. The origins of melatonin and its isomers can be traced back to photosynthetic bacteria and other primitive unicellular organisms. Since some of these bacteria are believed to be the precursors of mitochondria and chloroplasts these cellular organelles may be the primary sites of melatonin production in animals or in plants, respectively. Phylogenic analysis based on its rate-limiting synthetic enzyme, serotonin *N*-acetyltransferase (SNAT), indicates its multiple origins during evolution. Therefore, it is likely that melatonin and its isomer are also present in the domain of archaea, which perhaps require these molecules to protect them against hostile environments including extremely high or low temperature. Evidence indicates that the initial and primary function of melatonin and its isomers was to serve as the first-line of defence against oxidative stress and all other functions were acquired during evolution either by the process of adoption or by the extension of its antioxidative capacity.

## 1. Introduction

Melatonin is classified as a potent, naturally occurring antioxidant and a signaling molecule based on its primary and secondarily-evolved functions in organisms [1]. In animals, this molecule is formed exclusively from the essential amino acid tryptophan. Other organisms including bacteria, several protists, fungi, macroalgae, and plants, which possess the shikimic acid pathway, tryptophan can be synthesized starting with d-erythrose-4-phosphate and phosphoenolpyruvate, in phototrophs ultimately with carbon dioxide [2]. Therefore, melatonin production is not limited by tryptophan uptake in these species and can, thus, lead to remarkably high levels often exceeding those found in vertebrates by orders of magnitude [3,4,5].

**Figure 1:**
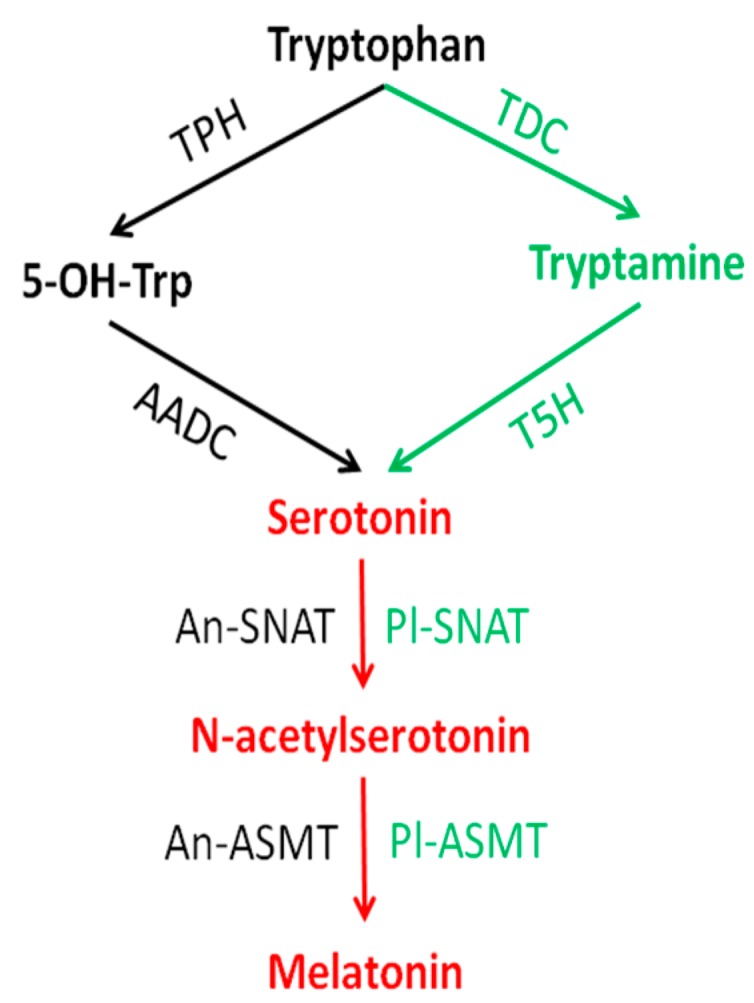
Melatonin synthetic pathway in animals and in plants. Black identifies the animal pathway, green indicates the plant pathway and red is their common pathway. TPH: tryptophan hydroxylase; AADC: aromatic l-amino acid decarboxylase; An-SNAT; animal serotonin *N*-acetyltransferase (the vertebrate enzyme is known as aralkylamine *N*-acetyltransferase, AANAT); An-ASMT: animal *N*-acetylserotonin-*O*-methyltransferase; TDC: tryptophan decarboxylase; T5H: tryptamine 5-hydroxylase; Pl-SNAT: plant serotonin *N*-acetyltransferase; Pl-ASMT: plant *N*-acetylserotonin-*O*-methyltransferase.

The synthetic pathway of melatonin in vertebrates has been intensively investigated and fully characterized [6,7]. This process starts with the initial precursor, tryptophan, and ends up with the final product, *N*-acetyl-5-methoxytryptamine, *i.e.*, melatonin. Four enzymes sequentially participate in melatonin biosynthesis. These include tryptophan hydroxylase (TPH), aromatic l-amino acid decarboxylase (AADC), serotonin *N*-acetyltransferase (SNAT) (official name in vertebrates: aralkylamine *N*-acetyltransferase (AANAT); for reasons of comparison with pathways in other organisms, we use here the abbreviation SNAT) and *N*-acetylserotonin *O*-methyltransferase (ASMT) (formerly hydroxyindole-*O*-methyltransferase (HIOMT)), respectively (Figure 1).

The genes for animal SNAT and plant SNAT share no homologies; this indicates their different origins during evolution.

SNAT usually is believed to be the rate-limiting enzyme of melatonin synthesis in vertebrates, although ASMT may limit this rate around the nocturnal melatonin maximum [8]. Under most circumstances SNAT activity is well coordinated with the environmental photoperiodic alterations to which the organisms are exposed. Thus, in the pineal gland of vertebrates, light inhibits and darkness enhances its activity, processes controlled by the circadian oscillator and by a photic shutoff mechanisms [9].

The photopigment, melanopsin, which is present in a small percentage of intrinsically photosensitive retinal ganglion cells, transduces select wavelengths (roughly 460–480 nm) into a neural message that is transferred through the retinohypothalamic tract to the suprachiasmatic nucleus (SCN). SCN is the master clock which regulates biological rhythmicity generally and also the circardian production of pineal melatonin. Information about the light/dark environment is sent from the SCN to the pineal gland via the autonomic nervous system.

The regulation of SNAT in the pineal gland, depends on the taxa examined. In many mammals including, e.g., frequently-studied rodents, its gene expression is either up or down regulated by the signals received from the SCN. The signaling molecule which activates the pinealocytes is the neurotransmitter, norepinephrine (NE); it is released from postganglionic sympathetic neurons whose cell bodies are located in the superior cervical ganglia. In the absence of light, these neurons are disinhibited and discharge NE into the synaptic cleft associated with pinealocytes. In turn, β and α_1_-adrenergic receptors on the pinealocytes are stimulated (for signal transduction pathways and modulation by other neurotransmitters see ref. [9]. In animals with a predominantly transcriptional control of SNAT, its mRNA levels are up-regulated up to 2 orders of magnitude [10]. In other species, especially primates and ungulates, SNAT is mainly controlled posttranslationally, by phosphorylation and dephosphorylation, and stabilization of the phosphorylated form by a 14-3-3 protein [11].

The neuronal transmission of light information as described for mammals represents a phylogenic apomorphism and has evolved as a consequence of the loss of extraretinal photoreceptors during a long-lasting period of nocturnalism in early mammals. Photoreception is a primary function of the pineal gland in non-mammalian vertebrates, such as agnathans [12,13,14], fish [15,16], amphibia [17], and sauropsids [16,18,19]. Melatonin formation and, often but not always, secretion is also demonstrable in other light-perceiving organs or tissues, such as parietal organ [19], retina [20,21,22,23], and deep brain photoreceptors [17]. Melatonin is easily released from frog eyecups, and the retina seems to be a major source of the circulating hormone in this group of animals [21,22]. In other vertebrates, the contribution of extrapineal melatonin to the amounts in the circulation is usually moderate or even very low. Despite overlying tissues, even in birds, the non-ocular photoreceptive tissues including the pineal gland of non-mammalian species are easily reached by light, which is perceived by specific visual pigments, such as pinopsin, parapinopsin and parietopsin or variants of them [19,24]. The light entry, at least in various species studied, is sufficient to control the activities of SNAT and melatonin production [25,26,27]. Photoreceptive cells and intracellular photoreceptive structures have been identified in the pineal glands of these species [15,28]. However, the signaling mechanisms from light perception to melatonin synthesis is not completely understood in extraretinal tissues, whereas this has been studied in some detail in the avian retina [29].

With regard to melatonin-producing tissues, a comparison between taxa also reveals a difference concerning SNAT (alias AANAT) subforms. In teleost fish, two subforms are present, AANAT1 in the retina and AANAT2 in the pineal gland. However, in all tetrapods including amphibia, only AANAT1 is present and expressed in various organs [17]. In frogs, its expression in the diencephalon was assumed to be related to melatonin formation in the deep brain photoreceptor [17]. The absence of AANAT2 in tetrapods should not be misinterpreted in terms of a loss, because teleosts are not the ancestors of tetrapods, which have instead evolved from the sarcopterygian clade, which is phylogentically distant to teleosts. In addition, many more subforms of SNAT have been identified in plants [30,31]

## 2. Melatonin Synthesis outside the Animals

The synthetic pathway of melatonin in microorganisms and in plants is quite different from that of vertebrates. In these organisms, the capacity to synthesize tryptophan has been retained, unlike in vertebrates. In addition, rather than tryptophan hydroxylation being the initial step in melatonin production, plants first decaboxylate tryptophan to form tryptamine and then hydroxylate tryptamine to form serotonin (Figure 1). The final two steps of melatonin biosynthesis in plants are similar to those in vertebrates (Figure 1); however, the animal homologues of SNAT and ASMT have not been detected in plants [32]. This is an indication of the multiple origins of melatonin synthetic enzyme genes in different phyla. However, the statements on plants are not valid for all phototrophs. In phototroph dinoflagellates, which have evolved by incorporating phototroph eukaryotic endosymbionts, the biosynthetic pathway of melatonin from tryptophan is identical to that in vertebrates. In the dinoflagellate *Lingulodinium polyedrum* (syn, *Gonyaulax polyedra*), tryptophan 5-hydroxylase was studied in some detail and found to differ from the vertebrate enzyme by using NADPH directly instead of a tetrahydropterin as a hydrogen donor [33,34], whereas it was blocked by the same inhibitors as in vertebrates [34,35]. However, the enzymes SNAT and ASMT revealed identical affinities to alternate substrates as known for vertebrates, findings that clearly indicated the usual sequential actions of SNAT and ASMT [36]. Pathway inhibition/metabolite supplementation studies confirmed the sequence of steps as well as the lack of rate limitation by aromatic l-amino acid decarboxylase [35].

Current data also indicate that the regulatory mechanisms in melatonin production may differ fundamentally between animals and plants. For example, in animals, light exposure uniformly suppresses melatonin production; thus, melatonin is referred to as the chemical expression of darkness [37]. In contrast, light exposure to plants under certain conditions in fact boosts melatonin production [38,39,40]. Currently, J. Kong *et al.* [40] (unpublished observation) have identified that in plants, gene expression of at least one ASMT isozyme is light-inducible. The positive correlation with light would be in accordance with the observation that plants living in habitats exposed to high light intensities, such as Mediterranean or alpine environments, usually have higher melatonin levels than same or related species in other locations [5,41,42]. However, apart from studies using different spectral light qualities [39], the light-associated rises can be poorly distinguished from high UV irradiation and light-induced stress affecting especially the photosystems. Moreover, increases of melatonin in response to light may not be the general rule in plants. As recently shown in *Oryza*, a heat-induced elevation of melatonin was antagonized by light [43].

The findings on light effects provide that signaling transduction pathways and regulatory mechanisms in vertebrates differ substantially from those in plants. Moreover, the major regulatory components of melatonin synthesis in mammals, *i.e.*, norepinephrine and its receptors, have not been detected in plants and are unlikely to exist. Another open question concerning the regulation of melatonin in plants is that of a circadian control. While the dinoflagellate *Lingulodinium* [44] and also numerous other microalgae [45] including chlorophyceans (like plants, members of viridiplantae), exhibit robust circadian rhythms of melatonin; this is less clear in the majority of plants studied. In a few cases, 24-h rhythms have been described in plants, e.g., in *Chenopodium* [46], which shows a nocturnal maximum and *Eichhornia* [40], with maximum around light/dark transition. Especially in species that have a melatonin maximum during or at the end of photophase, the alternative of circadian *vs.* light-controlled regulation has not been addressed. A direct upregulation of melatonin formation by darkness may also be possible and should, again, be distinguished from a circadian control. In rice seedlings, melatonin was reported to be enhanced by darkness of even of short duration, e.g., 1 h [43], findings supported by an enhanced ASMT expression in response to darkness [47]. However, based on what is being reported by us and others, We feel that studies on circadian melatonin rhythms in plants under artificial light may provide incorrect information because of the significantly low light intensity indoors compared to the natural sun light.

Considering the elevated free radical production that occurs during photosynthesis, it is easy to understand the advantage of increased melatonin production when plants are exposed to light, that is, high levels of melatonin are required to scavenge the large number of free radicals and preserve the photosynthetic function of plants. Many studies in fact have reported that exogenously-applied or endogenously-produced melatonin protects against chlorophyll degradation and preserves the photosynthetic function of plants due to a variety of stressors. Those stressors include drought [48], heat [49], cold [50,51], water-stress [52], extended darkness [53], herbicides [54] and soil pollutants [55]. These observations are consistent with the hypothesis that we originally proposed which is that as a naturally occurring potent antioxidant, melatonin will protect plants from a variety of abiotic stressors [56].

Another interesting phenomenon is that melatonin levels are much higher in plants than they are in vertebrates. In some species such as in *Glycyrrhiza uralensis* [39], cranberry [57] and several medicinal herbs [58,59] melatonin levels are several orders of magnitude higher than those in the serum of animals. Recently, unexpected high levels of melatonin have been identified in pistachio. Using a spectrofluorimetric assay compared with GC/MS analysis, Oladi *et al.* [60] reported that the levels of melatonin in the pistachio nut reached 230 µg/g. High levels of melatonin presumably are due to the fact that plants, unlike animals, cannot actively avoid environmental hazards because they are immobile. Thus, they require more protection from stressors by means of intrinsic mechanisms including high levels of the endogenously-produced antioxidant, melatonin [32]. Again, this speculation is supported by the observation that a variety of environmental insults induce a dramatic increase in melatonin levels in plants [55,61,62,63]. The characteristically stress-induced melatonin is not uniquely confined to plants but also has been seen in animals [64]. The ability to upregulate the protective molecule melatonin makes organisms more resistance to oxidative stress and environmental insults by enhancing their antioxidative capacity.

## 3. Melatonin Catabolism

In contrast to its synthesis, the metabolic pathways of melatonin are more complicated and require further clarification. It appears that two major principles are involved in melatonin metabolism. One is enzymatic catalysis and the other an enzyme-independent process. In the former, several enzymes have been identified. In the mammalian liver, primary enzymes for melatonin breakdown are cytochrome P_450_ isoforms (mainly CYP1A2, but also 1A1 and 1B1), which form, by monooxygenase activities, 6-hydroxymelatonin. The CYP isoforms 2C19 and 1A2 can also demethylate melatonin to its precursor, *N*-acetylserotonin. In the central nervous system, the same hydroxylation and demethylation reactions by CYP isoforms have been detected [7]. Additionally, melatonin can be deacetylated to 5-methoxytryptamine by aryl acylamidases, side activities of acetyl- and butyrylcholinesterases, or by a more specific melatonin deacetylase [7]. Another melatonin-metabolizing enzyme is indolamine 2,3-dioxygenase (IDO), which is present in microglia and macrophages and cleaves the pyrrole ring of the indolic moiety to give *N*^1^-acetyl-*N*^2^-formyl-5-methoxykynuramine (AFMK). However, the main substrate of IDO is not melatonin, but rather tryptophan [4]. Nevertheless, under conditions of microglia activation, e.g., by interferon-γ, the enzyme can contribute to melatonin degradation. The same reaction of pyrrole-ring cleavage is catalyzed by myeloperoxidase (MPO), eosinophil peroxidase (EPPO), and catalase (CAT) [65,66].

The metabolic products of enzymatic and nonenzymatic pathways overlap and, therefore, it is difficult to determine their involvement under specific conditions. Their common metabolic products include 6-hydroxymelatonin, AFMK and *N*^1^-acetyl-5-methoxykynuramine (AMK). 2-hydroxymelatonin is preferably formed by free radical-mediated hydroxylation. In mice and probably other species, a novel and unique product, *i.e.*, *O*-demethylated-6-hydroxymelatonin (=5,6-dihydroxy-*N*-acetylserotonin) is only generated via an enzymatic pathway [67]. Cyclic-3-hydroxymelatonin and nitrosated melatonin are believed to be generated exclusively by an enzyme-independent process during interactions of melatonin with a variety of ROS or reactive nitrogen species (RNS) [67,68,69,70,71,72]. Numerous products have been generated *in vitro* from AFMK and AMK by interactions with oxidizing radicals [73] or RNS [74], but their relevance *in vivo* remains to be demonstrated.

With the exception of dinoflagellates and yeast, the catabolism of melatonin in microorganisms and in plants is largely unknown. A melatonin metabolite identified in algae, protists and plants is AFMK [40,75]. In *Eichhornia crassipes*, a 24-h rhythm of AFMK was demonstrated for the first time [18,40]. AMK was formed in yeast cells incubated with its precursor AFMK, and an AMK metabolite, *N*-[2-(6-methoxyquinazolin-6-yl)ethyl]-acetamide (MQA), formed in the presence of an ammonium source, was released into the medium [76]. Deacetylation of melatonin to 5-methoxytryptamine (5-MT) is a major and physiologically important pathway in dinoflagellates, mostly studied in *Lingulodinium polyedrum*, but also in other species. 5-MT is, in this group of organisms, a potent inducer of asexual cysts, *i.e.*, resting stages to survive adverse environmental conditions [77,78], and also one of the strongest physiological stimulators of bioluminescence [79,80]. Temporally coincident circadian rhythms were demonstrated for the deacetylating enzyme, aryl acylamidase, and 5-MT [81]. In *Saccharomyces cerevisiae*, 5-MT was shown to be present under basal conditions and to be formed in high amounts from exogenous melatonin [82]. The presence of 5-MT was also reported for *Euglena gracilis*, for some rhodophyceans and the pheophycean *Petalonia fascia* [83]. Secondary metabolites formed from 5-MT are 5-methoxytryptophol (5-ML) and 5-methoxyindoleacetic acid (5-MIAA), compounds that can be also produced via other pathways from serotonin. However, in *Lingulodinium*, extensive formation of 5-ML from melatonin via 5-MT was shown under conditions of a precipitous melatonin breakdown that leads to cyst induction by 5-MT [3]. In the end, instead of 5-ML, 5-MIAA turned out to be the major end product of the deactylation pathway and to be released from the cells into the seawater [5,84]. It is anticipated that additional melatonin metabolites will soon be identified in yeasts, bacteria and plants since this is a rapidly-developing area of research. Herein, several fundamental issues related to the field of melatonin research in all domains of life will be addressed from an evolutionary point of view.

## 4. Origins of Melatonin

Experimental evidence suggesting the demonstration of the existence of melatonin can be traced back to 1917. At that time, McCord and Allen [85] documented that the bovine pineal gland contained a substance(s) which had the capacity to bleach pigmentation in amphibian skin. This substance was later isolated from the bovine pineal and structurally identified by Lerner and coworkers in 1958 [86]. The chemical structure of this substance was found to be *N*-acetyl-5-methoxytryptamine, commonly known as melatonin. Since melatonin was initially discovered in a mammalian pineal gland, at the time, it was believed to be exclusively produced in animals, particularly in pineal gland of vertebrates.

Subsequently, the retina and Harderian gland also were identified to produce melatonin [87,88,89]. Currently, evidence has accumulated that suggests that many animal tissues may be capable of producing melatonin [90]. In addition, melatonin was found in a unicellular organism, alga [91]. This discovery led scientists to consider the possibility that melatonin might have predated the evolution of vertebrates. Subsequently, melatonin was identified in primitive photosynthetic bacteria [92,93] including cyanobacteria [94,95], fungi [83], rhodophyceans [83], pheophyceans [96], and viridiplantae, including chlorophyceans [83] and embryophytes (land plants) [97,98]. Thus, the origin of melatonin was an estimated 2.5–3.5 billion years ago. That was the period in which the organisms were transitioning from anaerobic to aerobic metabolism, after water splitting by photosystem II had evolved in cyanobacteria (earliest records 3.5 billion years ago) [99].

During aerobic metabolism, organisms use oxygen as an electron recipient and in the terminal step, oxygen (O_2_) is reduced to water (H_2_O) after accepting four electrons. During this process, ROS are invariably generated due to the leakage of electrons from the electron transportation chain (ETC). When O_2_ accepts leaked electrons from the ETC, ROS are the result. It is estimated that up to 4%–5% of the O_2_ consumed by organisms during the aerobic metabolism eventually is reduced to ROS. High levels of ROS are toxic to cells and organisms. To protect against these toxins, organisms have developed simple and effective mechanisms to neutralize them, especially in primitive life forms such as in bacteria and other unicellular organisms. Melatonin probably appeared initially as an antioxidant and free radical scavenger. During later evolution, unicellular organisms evolved into complex multi-cellular organisms, including vertebrates; however, the structure of melatonin has never changed. Accordingly, the optimal structure of melatonin as a simple and potent antioxidant requires little modification.

Based on the theory of endosymbiosis as proposed by Sagan [100] and other evidence, we hypothesized that the melatonin’s synthetic trait in eukaryotic cells might have been horizontally transferred from prokaryotic cells, particularly, from bacteria [101]. For example, as mentioned previously, the primitive photosynthetic alpha-proteobacteria, particularly, *Rhodospirillum rubrum*, might be phylogenetically the oldest organisms which have the capacity to synthesize melatonin [92]. Alpha-proteobacteria are currently accepted as being the precursor of mitochondria [102]. The endosymbiotic theory states that when the proto-eukaryotic cells (having nuclei but no mitochondria) endocytized alpha-proteobacteria related to *Rhodospirillum rubrum*, rather than digest this bacterium they developed a symbiotic association with them. The host cell provided resources to this bacterium and, due to its high efficiency for oxidative-phosphorylation, the bacterium rewarded the host cell with abundant energy in the form of ATP. The majority of bacterial genes were either deleted or transferred to the nucleus, while others, primarily proto-eukaryotic genes, became involved in the structure and function of the former endosymbionts, the mitochondria [103].

Due to the high level of ROS generated by mitochondria, it is not surprising that the ability to synthesize melatonin was conserved by the host cells. We have speculated that mitochondria are the original site of melatonin production in eukaryotic cells and they may still possess this capacity [101,104]. If this is verified, virtually every cell, tissue and organ may be equipped with the melatonin synthetic machinery since they all contain mitochondria, although the quantities probably formed vary considerably. The expression of the genes involved is certainly controlled in a cell-specific manner, and, with regard to the completely known mtDNA sequence of higher vertebrates, which contain only a small number of mitochondrially-encoded genes, *SNAT* and *ASMT* genes are, at least, in all animals studied, located in the nucleus. More and more sites of extrapineal melatonin formation have been detected in vertebrates [105], which would be in line with a more abundant ability to form the methoxyindole. However, the mitochondrial import of SNAT or ASMT proteins remains to be demonstrated. Whether mitochondria of more primitive or phylogenetically distant eukaryotes have retained the genes for melatonin biosynthesis is unknown and should be clarified.

The situation is different in phototrophic organisms. Cyanobacteria are known to be capable of producing melatonin. The *SNAT* gene of a *Synechocystis* strain has been cloned [106]. These bacteria are regarded as the precursors of chloroplasts. In rice, SNAT was shown to be localized in chloroplasts, whereas ASMT is present in the cytosol [107]. Thus, chloroplasts contribute to melatonin synthesis in plants. Whether this is also valid for eukaryotic phototrophs other than plants, and for plants generally, remains to be studied. With regard to the fact that chloroplasts produce much higher amounts of ROS than mitochondria [108], and to the critical role of ROS in photoinhibition, especially the appropriate functioning of photosystem II [109], the antioxidative capacity of phototrophs may be considerably higher than that of heterotrophs. Indeed, as mentioned previously, plants frequently have much higher melatonin levels than do animals.

## 5. Emergence of Melatonin Isomers

**Figure 2:**
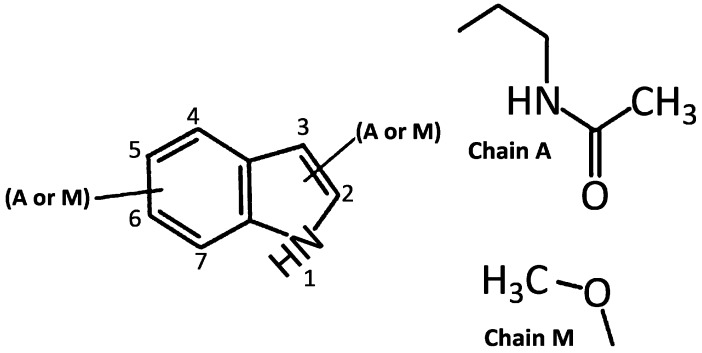
Chemical structures of proposed melatonin isomers. The numbers identify the positions on the indole ring and the A or M represent the side chain A and side chain M, respectively. When A is located at position 3 and M at position 5, the molecule is melatonin.

For more than five decades after the identification of melatonin [86], it was considered to be the only naturally occurring acetylmethoxyindoleamine in organisms. The initial publication referring to naturally-occurring melatonin isomers appeared in 2011 [110]. These isomers contain the same number of atoms as melatonin but have different arrangements of their atoms in space. All melatonin isomers are structural isomers and their functional groups are joined at different sites on the indole nucleus. Theoretically, based on the possible positions of the two side chains on the indole nucleus of melatonin (only seven positions are available for occupation by the side chains), it has been calculated that as many as 42 melatonin isomers (6 × 7) could exist (Figure 2).

A nomenclature of these melatonin isomers has been proposed [111]. In the last two decades, several synthetic melatonin isomers were produced and their binding properties to melatonin membrane receptors and their interactions with ROS were tested [112,113,114]. Although the properties of these synthetic melatonin isomers were similar to those of melatonin, the authors of these reports did not speculate that these isomers may also be naturally-occurring in organisms.

The commonly-used methods for melatonin identification during this interval included radioimmunoassay, ELISA and HPLC. None of these methods, however, had the capacity of identifying melatonin isomers. Although several studies had used mass spectrometry (MS) to detect melatonin in biological samples, these publications do not mention the presence of isomers [115,116,117,118]. Using HPLC with mass/mass spectrometry (HPLC-ESIMS/MS), Rodriguez-Naranjo *et al.* [110] found that a supposed melatonin fragmentation of *m*/*z* 233 had the uncharacteristic minor fragments of 196, 161, and 141 which did not coincide with those of the melatonin standard. Upon detailed analysis, they identified that these molecules were actually isomers of melatonin. Subsequently, several reports have confirmed that melatonin isomers are indeed present in wine [119,120,121] and also in fermented orange juice [122].

The distribution of melatonin and its isomers among wine products varies substantially. Some brands of wine contain only melatonin, some only contain isomers and others contain both melatonin and its isomers [121]. These studies also revealed that neither melatonin nor its isomers in wine are derived from the grapes *per se*, but rather they were formed during the process of fermentation [120]. Thus, melatonin and melatonin isomers appearing in wine are mainly derived from yeasts and bacteria. Based on these observations, we have predicted that many important fermented products including bread, yogurt, cheese and kimchi, will have relatively high levels of melatonin and its isomers [111].

Melatonin and its isomers have been recently identified in some of these fermented products, for example, in bread, yogurt, beer and kefir [123,124]. Meanwhile, melatonin isomers have been also identified in plants and plant products. These include walnut, olive, cherry, tomato, coffee, cacao and black and green tea [124]. These are nutritional products widely consumed by humans and the beneficial effects of melatonin and its isomers from these sources could be substantial [125]. Another noteworthy observation is that the levels of melatonin isomers in wine, in bread and in plant products are up to one thousand times higher than those of melatonin [120,123,124]. This raises a critical question as to what is the functionally and nutritionally dominant acetylmethoxyindole in microorganisms and in plants, *i.e.*, is it melatonin or its isomers? Since the research on melatonin isomers is in its infancy, based on current information no definite claims can be made. However, judging from the much higher levels of melatonin isomers than those of melatonin, the isomers may play very important roles which could exceed those of melatonin. In addition, the nutritional or medicinal values of high levels of melatonin isomers in fermented products and plants are also a new area awaiting exploration.

The origins of melatonin isomers appear as old as melatonin *per se* since these isomers have been identified in unicellular organisms (fungi, bacteria) and plants as mentioned above. Especially in plants, several isoforms of SNAT and ASMT have been identified, for example, in apple [30] and rice [126]. The activities of some of these isoforms do not always correspond to the production of melatonin. Whether some isoforms of SNAT and ASMT are actually the enzymes for the synthesis of melatonin isomers is currently unknown. There is no report as to the functions of melatonin isomers in organisms. It is our speculation that the isomers may exhibit similar functions to those of melatonin. Thus, they can be complementary to each other when a mutation has occurred in the synthetic pathways of melatonin. This would allow organisms to avoid catastrophic consequences due to the deficiency of melatonin synthesis when they face extensive environmental insults or oxidative stress, situations in which they would normally be protected by melatonin.

No evidence currently exists showing that melatonin isomers are present in animals. From an evolutionary point of view, animals might also have the capacity to produce these isomers. The failure to detect them in animals could be either due to the limitation of the currently used assay methods or, alternatively, this capacity may have been lost or completely replaced by synthesis of melatonin during evolution. It is anticipated that melatonin isomers will be detected in animal tissues including in humans. It is estimated that around 10^14^ bacterial cells are present in the gut and many beneficial effects for the host derive from these inhabitants [127]. As already mentioned, microorganisms including yeast and bacteria produce melatonin isomers. It has been reported that the host and microorganisms co-metabolize indolamines including melatonin [128] and perhaps its isomers. The host and microorganisms frequently exchange materials. A recent clinical trial showed that male patients who took a probiotic which contained viable lyophilized bacteria including *Bifidobacterium* (*B. longum*, *B. infantis* and *B. breve*); *Lactobacillus* (*L. acidophilus*, *L. casei*, *L. delbrueckii* ssp. *bulgaricus* and *L. plantarum*) and *Streptococcus* (*salivarius* ssp. *Thermophilush*) had significantly higher morning salivary melatonin level than that of control subjects [129]. These bacteria have previously been industrially used to generate melatonin for consumers [111]. It seems likely that melatonin isomers generated in gut flora would be absorbed by the host organism. Obviously, the biophysiological impact of this exchange of melatonin isomers between the host and microorganisms is currently unknown.

A major shortcoming for the study of melatonin isomers is the isolation methodology. As noted, structurally, there are potentially 42 melatonin isomers. Currently, only a few have been structurally deduced from the spectra obtained from MS analysis. To structurally identify isomers, NMR or X-ray diffraction spectrum must be performed on the isolated isomers. An alternative would be to synthesize the isomers and use them as standards to compare with isolated isomers using HPLC and MS. With improvements in methodology, additional melatonin isomers may be structurally identified in microorganisms, fermented products, plants, plant products and also in animals.

## 6. Phylogenic Traits of the Melatonin Synthetic Enzyme, SNAT

All species from primitive photosynthetic bacteria to the most complex multi-cellular organisms including mammals produce structurally-identical melatonin; however, the enzymes for melatonin synthesis, particularly the rate-limiting enzyme SNAT, are probably not genetically associated. For example, the homologue of the SNAT (AANAT) gene in vertebrates is not present in plants [32]. This raises a question as to the phylogenic origin of the enzymes required for melatonin synthesis, especially the origin of the presumed rate-limiting enzyme SNAT in different species during evolution. A horizontal gene transfer hypothesis was proposed to explain this phenomenon [128,130], for example, the SNAT gene did not vertically pass from ancestors to their descendants but rather this gene was horizontally transferred from gram positive bacteria to other species. This hypothesis only partially explains the widely different genes encoding the melatonin synthetic enzymes in organisms.

**Figure 3:**
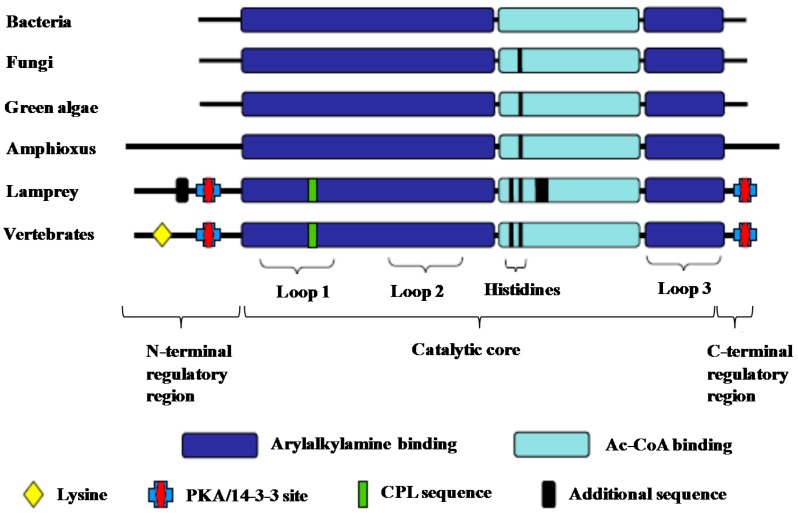
Structural evolution of serotonin *N*-acetyltransferases (SNATs) among species (From Falcon *et al.* [12]). The major structural differences in SNATs among phylogenetically distant species are the regulatory regions; however, the catalytic cores of these enzymes share some similarity. The term vertebrates in Figure 3 refers more correctly to gnathostome vertebrates.

At least, for the SNAT genes in vertebrates, they were not horizontally transferred from bacteria but evolved from the non-vertebrate form of SNAT after the Cephalochordate–Vertebrate split over one-half billion years ago [12]. This speculation implies that the SNAT gene was vertically inherited from phylogenic ancestors and the DNA sequences as well as the structural differences may be an adaptation caused by natural selective forces in different species during evolution. The most striking differences between the vertebrate’s SNAT and SNATs from other phylogenic species are found in regulatory regions of the encoded proteins, particularly in the *N*-terminal and in the *C*-terminal regulatory regions. However, the catalytic regions share some similarity (Figure 3). The authors speculated that the differences of SNATs in a variety of species exhibit different metabolic roles, *i.e.*, the function of the vertebrate SNAT is to synthesize the signaling molecule, melatonin, while the non-vertebrate SNAT is used to detoxify aralkylamines, to acetylate polyamines (in *Branchiostoma*) [129,131], and to participate in integument sclerotization (in insects) [130,132]. However, in insects, especially diptera, the situation cannot be easily judged because of multiple SNAT (AANAT) isoforms, such as AANAT1a, AANAT1b and AANAT2 in *Drosophila melanogaster* [130], and additional subforms, such as AANAT5b and AANAT7 in *Aedes aegypti* [131,133]. Based on available evidence, all non-vertebrates included in Figure 3, *i.e.*, bacteria, fungi, green algae and the cephalochordate *Branchiostoma*, produce melatonin [83,132,134].

In spite of the different structures of SNAT between vertebrates and non-vertebrates, these enzymes share similar catalytic kinetics for melatonin synthesis, as far as they are really involved in this reaction and not in the acetylation of other molecules such as polyamines. Therefore, it might be of interest to test the possibility that SNAT of non-vertebrates not only functions in the detoxification of aralkylamines as suggested by Falcon *et al.* [12] but may also be involved in the synthesis of melatonin isomers. Melatonin has been shown to be produced in various taxa of invertebrate animals [133,135]. In these considerations, the situation in *Drosophila* should be left apart, because the precursor *N*-acetylserotonin (NAS) is, in this organism, three orders of magnitude higher than melatonin [136], because of an obvious loss of ASMT, which is poorly replaced by another, less specific methyl transferase(s), whereas other insects do express ASMT [137]. SNAT as an NAS-synthetizing enzyme in the absence of further *O*-methylation to melatonin may not be that uncommon and has been even considered in mammalian tissues, e.g., brain regions [138]. With regard to the widespread occurrence of melatonin in invertebrate animals, its synthesis may be the primary function of one of the SNAT isoforms, whereas other isoforms may have specialized into functionally other directions. However, this assumption remains to be directly demonstrated. Moreover, it should be tested whether SNAT isoforms are involved in the formation of melatonin isomers. It will be important to determine whether (i) the isomers derive from melatonin by subsequent enzymatic or nonenzymatic isomerization; (ii) precursors of melatonin are isomerized; or (iii) some isomers derive from serotonin hydroxylation in positions other than ring atom 5.

Another extreme example of phylogenetic divergence is, as mentioned previously, the lack of SNAT gene homologues common to vertebrates and plants. The characteristic of a vertically-inherited feature of SNAT cannot be applied to these phylogenically different clades. Thus, current hypotheses which are used to explain the phylogenic origins of the melatonin synthetic machinery (synthetic enzymes) are not sufficiently clarified and may require further modification.

The evidence is compelling that in the earliest stages of evolution melatonin is already present, as are its synthetic enzymes and these enzymes seem to have multiple origins. For example, the melatonin biosynthetic machinery differs between the alpha-proteobacterium, *Rhodospirillum rubrum*, and cyanobacteria. This is not unreasonable since these species evolved under completely different environmental conditions and they faced different natural selective processes. Adapting for survival in different environments would likely lead to different structures of their melatonin synthetic machineries.

Analyses of DNA sequences and protein residues have revealed that the SNAT gene in plants (rice) is the homologue of cyanobacteria and they share 56% amino acid homology [106]. This observation strongly implies that the SNAT gene in plants derived from SNAT gene of cyanobacteria. The homologue of the plant SNAT gene is not present in other taxa. Thus, it appears that the SNAT genes of other eukaryotic organisms including fungi, invertebrates and vertebrates may have evolved from *Rhodospirillum rubrum* or closely related species (the presumed precursor of mitochondria), since their SNAT genes share some similarity (Figure 3).

The transfer of bacterial SNAT to these species probably occurred as a result of endosymbiosis. After the endosymbiotic bacteria gradually transformed either into mitochondria or chloroplasts, the SNAT genes of these bacteria were finally incorporated into the nuclear genome of each host. Currently, there is evidence suggesting that the SNAT protein in pinealocytes of vertebrates is exclusively located in mitochondria [139,140]. Also, SNAT has been identified and localized exclusively in the chloroplasts of plants [107]. These observations further support the hypothesis of the different origins of the melatonin synthetic enzymes as a result of endosymbiosis. The SNAT genes were subsequently modified by mutations and in response to natural selective pressures in different species during evolution. This process may explain the wide variety of phylogenic traits of melatonin biosynthesis and particularly, the different structures of their melatonin synthetic enzymes in different species.

## 7. Melatonin and Its Isomers in Archaea?

Different from the previous five kingdoms, modern biology classifies all organisms into three domains which include bacteria, archaea and eukaryotes. Melatonin is found in bacteria and eukaryotes. Melatonin isomers have also been identified in these two domains. We presume that melatonin and its isomers would be a universal phenomenon. To date, however, there are no reports of melatonin and its isomers in archaea.

It has been reported that the archaeal basal transcription apparatus is similar to that of eukaryotes rather than to that of bacteria, although their morphologies superficially resemble those of bacteria [141]. It was hypothesized, therefore, that archaea may be the direct precursors of eukaryotes [142] or eukaryotes might be the result of fusion between bacteria and archaea [143]. Some archaea can survive in extreme environmental conditions such as elevated temperature, high pH and high salinity [144,145]. Many archaea also inhabit the gastrointestinal tract of animals including that of the human [146,147]. The importance of archaea in ecological systems [148] and also to human health and disease [149,150], particularly their relation to obesity [151,152], has drawn much attention recently. Even though no direct evidence documents whether archaea produce melatonin and/or its isomers, the melatonin synthetic capacity may exist in archaea. However, such considerations have to give attention to two facts. First, the majority of archaea are extremophils, among them numerous anaerobic species, which are usually not challenged by ROS but may face other extreme environmental insults that would generate other reactive molecules. Thus, it is important to determine whether these species also synthesize melatonin. Second, horizontal gene transfer occurs also between bacteria and archaea, largely depending on a common microhabitat [153,154]. Examples are the presence of archaeal homologs of kai proteins, the bacterial circadian oscillator components [155], and the presence of bacterial genes in the archaeon *Methanosphaera stadtmanae*, a species present together with high numbers of anaerobic bacteria in the human gut [153]. Therefore, even if archaea might not have invented a melatonin biosynthetic pathway (which is easily possible), they might have acquired it by horizontal gene transfer from bacteria.

From an evolutionary point of view, archaea evolved in parallel with bacteria and they are believed to have played a pivotal role in the emergence of the eukaryotes [143,156]. If both bacteria and eukaryotes have the capacity to synthesize melatonin and melatonin isomers, there is reason to suspect that archaea either evolved or acquired the capacity to synthesize melatonin and/or its isomers. A hypothetical acetyltransferase has been cloned, purified from a thermophilic archaeon (*Pyrococcus furiosus*) [157]. The archaean acetyltransferase belongs to the GCN5-related *N*-acetyltransferase (GNAT) superfamily. One member of the GNAT superfamily has been cloned and functionally characterized in the cyanobacterium (*Synechocystis* sp. PCC 6803) [106]. This enzyme fully functions as a SNAT to synthesize *N*-acetylserotonin from serotonin. The presence of GNAT members in both bacteria and archaea strongly indicates the occurrence of inter-domain horizontal gene transfer of, at least, a GNAT ancestor gene.

Whether the GNAT in archaea functions as the GNAT of cyanobacteria remains unknown; however, this possibility cannot be excluded. In addition, as noted, some archaea inhabit extremely harsh environments (100 °C, pH 1–2) [158,159]. They require protection of their DNA and proteins from these environmental stressors. They often also share their habitats with extremophil bacteria. Melatonin is a proven, strong protector against such harsh environments, particularly heat, in algae and plants [49,61,160]. As in other organisms, for their survival, archaea may have taken advantage of a melatonin synthetic capacity. This possibility is supported by recent observations. Back *et al.* [31,107] have observed that the penultimate enzyme in melatonin synthesis, SNAT, and also the last enzyme, ASMT, in a plant (rice) tolerate extremely high temperatures and pH (pH optimum of SNAT at 8.8). The activities of both enzymes at a temperature of 95 °C are much greater than those at 25 °C, which is a suitable growth temperature for rice. This temperature tolerant trait of plant SNAT and ASMT is similar to that of some cyanobacteria; again; this provides further evidence as to the origin of the plant melatonin synthetic enzymes from cyanobacteria.

**Figure 4:**
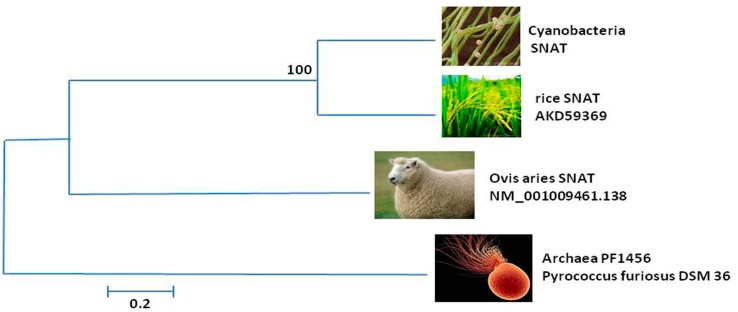
The phylogenetic tree of SNATs in different species. The homologous genes of SNATs in cyanobacteria, rice, Ovis aries and archaea were blasted in NCBI. The phylogenetic tree was constructed using the neighbor-joining method and a bootshrap test with 1000 iterations, using MEGA5.2 software. The scale represents the number of nucleotide changes during evolution. The GenBank accession numbers are NP_442603 (Cyanobacteria SNAT), AK059369 (Rice SNAT), NM_001009461.1 (Ovis aries SNAT), NP_579185.1 (Archaea PF1456 Pyrococcus furiosus DSM 3638 SNAT). The results indicated that the cyanobacterial SNAT gene is more closely related to that of rice.

Another question related to the temperature tolerance of those enzymes is whether they originated *de novo* in cyanobacteria or whether this trait was horizontally transferred from the thermophilic archaea to cyanobacteria, or *vise versa*. To answer this question, homologues of SNAT genes from cyanobacteria, rice, sheep and archaea, representatives of each of the three domains were analysized (Figure 4).

This showed that the sequence of amino acid residues in cyanobacteria and rice are the most closely related and suggest that the rice SNAT gene may have been endosymbiotically derived from the cyanobacterium. It was unexpected that cyanobacterium and rice SNAT genes would be more closely related to animals (sheep) than to archaea (Figure 4). This implies that the thermophilic SNAT genes of cyanobacteria and rice may not have any association with the thermophilic SNAT gene of archaea. Therefore, one might conclude that the SNAT genes of cyanobacteria and archaea have evolved independently when they encountered similar high environmental temperatures. However, this conclusion does not consider the evolutionary velocity under extremophil conditions nor the comparison with a non-extremophil cyanobacterium. Additional investigations are required to clarify the real associations among the different domains regarding their genes for melatonin synthesis.

## 8. Functional Evolution of Melatonin and Melatonin Isomers

Since its discovery, numerous functions for melatonin have been defined [161]. These include as a signaling molecule to transduce photoperiodic information, regulating the seasonal reproductive activity in photoperiodic animals, facilitating sleep physiology, promoting immunoresponsiveness, balancing energy metabolism, suppressing carcinogenesis, promoting stem cell proliferation, anti-inflammation, scavenging free radicals and retarding aging [162,163,164,165,166,167,168,169,170,171,172,173]. Some of these functions are mediated by melatonin receptors (membrane and nuclear) [174,175,176,177,178] while others are receptor-independent [65,179].

It seems unlikely that melatonin simultaneously acquired so many functions at its initial presence in species, especially in unicellular organisms. Melatonin is a phylogenetically old molecule with an estimated age of 2.5–3.0 billion years. It likely first appeared in primitive photosynthetic bacteria. The majority of melatonin’s functions as mentioned above including sleep promotion, inhibition of carcinogenesis and enhancement of immunoresponsiveness definitely cannot be applied to bacteria or other unicellular organisms. It is obvious that these functions of melatonin were acquired during evolution [1].

As to the primary and initial function of melatonin, it has been hypothesized that melatonin served as a free radical scavenger and antioxidant to protect organisms from internal and external insults, *i.e.*, oxidative stress [1,80]. Free radical reactions which lead to oxidative stress are one of the most basic cellular processes that occur in organisms from primitive photosynthetic bacteria to humans. Melatonin is a highly effective and functionally diverse molecule combating free radical reactions [65]. How did melatonin from an exclusively antioxidant molecule in primitive bacteria develop to be a pleiotropic molecule in multicellular organisms? For example, what are the evolutionary steps in the derivation of melatonin into a photoperiodic signaling molecule? This may be also related to its free radical scavenging activity. In photosynthetic bacteria such as the cyanobacterium, more free radicals are produced during the photophase than during the scotophase due to their photosynthetic activity [180,181,182]. If we assume that these bacteria produce melatonin at a constant rate throughout a light/dark cycle, then larger amounts of melatonin would be consumed during the detoxification of excessively-produced free radicals during the day. As a result, the differential consumption of melatonin would result in higher levels of melatonin at night than during the day, *i.e.*, a rhythm reflecting its metabolism rather than its synthesis. As unicellular organisms, phototroph bacteria can directly perceive photoperiodic changes and synchronize their biological activities accordingly. When unicellular organisms evolved into complex multicellular organisms, the cells of multicellular organisms could no longer respond directly to the photoperiodic changes, they required the photic information to be transduced into a signaling molecule that could travel throughout the organism to synchronize cellular activities. Since melatonin levels have already passively reflected the photoperiodic changes of light/dark cycle in their ancestors (photosynthetic bacteria), multicellular organisms may have adopted the melatonin cycle as a signaling system for this purpose. In contrast to their ancestors, *i.e.*, unicellular organisms, multicellular organisms actively increase pineal melatonin production during the night which serves as a circadian signal to all other cells [1].

For a number of mammals, their reproductive capability fluctuates seasonally and this closely correlates with the changing photoperiodic information [183]. Deer, hamsters and goats, *etc.* are classified as photoperiod-dependent seasonal breeders [184,185,186,187,188]. The annual rhythm in reproductive performance is critical to survival of species, since young born at inappropriate times, e.g., winter, would suffer high mortality which could compromise propagation of the species [189]. Thus, they adopted the seasonal alterations in the duration of the nocturnal melatonin message, *i.e.*, the prolonged peak of melatonin in winter and short peak in summer, to synchronize their annual cycle of reproduction.

As to melatonin’s relationship to sleep physiology, vertebrates utilize the melatonin rhythm primarily as a dark/light signal rather than as a soporific agent suitable for sustaining sleep maintenance. For example, in diurnally-active animals, a high nocturnal level of melatonin signal indicates that it is the time for sleep. As a result, high levels of melatonin in diurnal animals promote sleep, mainly in terms of sleep initiation via (i) SCN-dependent control of the hypothalamic sleep switch [105,190] and (ii) thalamic effects that lead to sleep spindles [191], whereas sleep duration and maintenance are only poorly affected [192] In contrast, in nocturnal animals, the nightly high levels of endogenous melatonin are not required for sleep [193] and may instead signal the time of forage or other activities. The differential functions of melatonin on sleep physiology in different species further prove that many functions of melatonin were gradually acquired during evolution [1].

Since melatonin receptors, particularly membrane receptors, appeared presumably after the evolution of unicellular organisms (there are no reports of melatonin receptors in unicellular organisms), receptor-mediated functions of melatonin were likely acquired at different later stages of evolution. They are secondary to the function of melatonin as a free radical scavenger. Nevertheless, intracellular effects of melatonin beyond radical scavenging do exist in unicells and, therefore, pathways of signal transduction must be present. In *Lingulodinium*, melatonin was shown to strongly upregulate aryl acylamidase [81], to downregulate diurnally peaking enzymes such as tryptophan 5-hydroxylase and superoxide dismutase [81,83] and to modulate glutathione S-transferase [83]. Using standard procedures of 2-^125^I-melatonin binding, no melatonin receptors were detected in this organism [194]. However, regulated binding sites may exist, because, in the presence of 1 µM exogenous melatonin, the indoleamine accumulated up to 25-fold within 3 h and declined thereafter, despite an unchanged extracellular concentration [195].

Other actions of melatonin are logically the expanded spectrum of its antioxidant activity. These actions include retarding some age-related process [166,196,197,198,199,200], its anti-inflammatory actions [201,202,203,204], its ability to resist neurodegenerative changes [205,206,207], the prevention of apoptosis in normal cells [208,209,210,211] and the preservation of mitochondrial and chloroplast physiology [52,212,213,214]. These actions relate to some aspects of free radicals and oxidative stress. Many studies have unambiguously proven that the ability of melatonin to modulate these processes relate, at least in part, to the free radical scavenging and antioxidative action of melatonin. In addition to the direct free radical scavenging activity, melatonin activates a variety of antioxidant enzymes [215,216,217] while it suppresses some prooxidant enzymes [218,219]. These stimulatory and inhibitory effects are referred to as the indirect antioxidant activities of melatonin. The indirect antioxidant activity of melatonin is mediated by ether melatonin membrane receptors or its nuclear binding sites [220,221,222]. An exception is the relationship between melatonin and quinone reductase 2 (QR2, E.C. 1.10.99.2). Initially, this enzyme was classified as the melatonin membrane receptor 3 (MT3) [223]. Nosjean *et al.* [224] identified that MT3 is not a melatonin membrane receptor but it is QR2. Melatonin directly binds to this enzyme to produce its activity rather than via a receptor-mechanism [225,226]. Based on available data, Tan *et al.* [227] hypothesized that melatonin might be a co-factor of QR2. The results from a subsequent report did not fully support this hypothesis [228]. QR2 is considered a second phase enzyme. Melatonin’s binding to the catalytic site of QR2 inhibits its activity and this inhibition may play an important role in ROS generation and detoxification [229]. The exact mechanisms and biological consequences of melatonin binding to this enzyme are currently unknown. Some of the indirect antioxidant activities of melatonin may be mediated by its binding to QR2.

## 9. Concluding Remarks

In the current review, several fundamental questions related to melatonin have been discussed from the point of view of evolution. Melatonin is an ancient molecule that appears in primitive photosynthetic bacteria. The initial and primary function of melatonin likely relates to its free radical scavenging and antioxidant activities. Other functions of melatonin were theoretically acquired during evolution either by the process of adoption or by the extension of its antioxidative capacity. The existence of melatonin isomers in organisms also raises fundamental questions as to which of these are the predominant functional products and which are present in greatest abundance. In fermented products including wine and bread, as well as in some plants, the levels of melatonin isomers are much higher than those of melatonin. Thus, biological functions of naturally occurring melatonin isomers in species appear to be likely and may extend to nutritional values for their consumers. The capacity to synthesize melatonin and/or its isomers seems to be a universal phenomenon in organisms. Thus, we speculate that in addition to bacteria and eukaryotes the domain of archaea should also have the ability to biosynthesize melatonin and its isomers. This would aid in their survival in the extremely harsh environments such as in excessively high temperatures. Melatonin has been shown to protect species, particularly algae and plants, from harsh environmental insults.

The phylogenic features of melatonin’s synthetic genes are complex. For example, no homologous genes for SNAT and ASMT have been identified between plants and animals. In addition, the DNA and protein structures of a hypothetical SNAT gene occurring in thermophilic archaea is quite different from the SNAT gene of cyanobacteria although this SNAT of cyanobacteria is heat resistant. Collectively, it appears that the genes for melatonin’s synthetic enzymes have had multiple independent origins. At this point, the details of these evolutionary processes are obviously not available. To date, the accumulated evidence suggests that in plants the genes for melatonin synthesizing enzymes derived from the cyanobacteria and in animals they derived from a purple non-sulfur bacterial species, particularly, *Rhodospirillum rubrum* or a related ancestor*.* Cyanobacteria are believed to be the precursor of chloroplasts and alpha-proteobacteria like *Rhodospirillum rubrum* are proposed as the precursor of mitochondria. Thus, the genes for the melatonin synthetic enzymes in eukaryotes were presumably acquired from bacteria during the process of endosymbiosis. This implies that chloroplasts and mitochondria may be the primary sites for melatonin synthesis eukaryotic cells.
